# Night-shift work and susceptibility to infectious diseases: a systematic review and meta-analysis

**DOI:** 10.5271/sjweh.4225

**Published:** 2025-07-01

**Authors:** Bette Loef, Esmee Bosma, Linda W M van Kerkhof, Karin I Proper, Debbie van Baarle, Martijn E T Dollé

**Affiliations:** 1 Center for Prevention, Lifestyle and Health, National Institute for Public Health and the Environment, Bilthoven, The Netherlands.; 2 Center for Health Protection, National Institute for Public Health and the Environment, Bilthoven, The Netherlands.; 3 Department of Public and Occupational Health, Amsterdam UMC, Vrije Universiteit Amsterdam, Amsterdam Public Health research institute, Amsterdam, The Netherlands.; 4 Center for Infectious Disease Control, National Institute for Public Health and the Environment, Bilthoven, The Netherlands.; 5 Department of Medical Microbiology and Infection Prevention, University Medical Center Groningen, University of Groningen, Groningen, The Netherlands.

**Keywords:** infection, night work, respiratory infections, SARS-CoV-2, shift work

## Abstract

**Objectives:**

A growing body of research on infection susceptibility among night-shift workers has emerged, particularly since the COVID-19 pandemic. However, a comprehensive overview is still lacking. Therefore, this review aimed to synthesize the evidence on the association between night-shift work and susceptibility to infectious diseases.

**Methods:**

Embase and PsycINFO were systematically searched for studies published up to September 2024. Studies were included if they comprised a working population, night-shift workers were compared to non-shift workers, and the outcome was an infectious disease. Results were descriptively synthesized for common respiratory infections (flu and common cold), SARS-CoV-2 infection, and other infections. Pooled effect estimates were calculated using random-effects meta-analysis.

**Results:**

In total, 16 articles describing 14 studies among 191 320 workers were included. Based on 4 studies, night-shift work was not associated with a significantly increased risk of common respiratory infections [odds ratio (OR) 1.11, 95% confidence interval (CI) 0.97–1.27, I^2^=65.8%]. However, night-shift workers had a higher risk of SARS-CoV-2 infection than non-shift workers (OR 1.31, 95% CI 1.09–1.58, I^2^=92.2%, N=10 studies). This association was stronger in higher-quality studies and studies conducted in the first year of the COVID-19 pandemic. For other infections, insufficient studies were available to conduct a meta-analysis. The certainty of evidence was graded very low due to a limited number of (prospective cohort) studies and high inconsistency in the available studies.

**Conclusions:**

This systematic review and meta-analysis showed that night-shift work was associated with an increased risk of SARS-CoV-2 infection, but not of common respiratory infections. To address the lack of high-certainty evidence, more studies are needed that apply a prospective design with appropriate adjustment for confounding factors and more extensive information on night-shift work exposure.

In today’s society, workplaces have increasingly moved toward a 24/7 economy, ensuring round-the-clock availability of goods and services (1). However, this shift also means that many work schedules include non-standard work hours. As a result, a substantial part of the labor force works during the night (2, 3). Yet, night-shift work is associated with many adverse health effects, including an increased risk of cardiovascular diseases and diabetes mellitus type 2 (4–6). Circadian rhythm disruption and disturbed sleep are underlying mechanisms that are linked to the development of these health problems among night-shift workers (7, 8).

Because of this circadian rhythm disruption and disturbed sleep, night-shift workers may also be exposed to an elevated risk of infectious diseases. The circadian rhythm influences various immune responses, which may be adversely affected when the circadian rhythm is disrupted (9). Furthermore, disturbed sleep has been found to affect the immune system and increase infection risk (10, 11). In the last decade, more research has been devoted to examining whether night-shift work indeed negatively impacts the functioning of the immune system and increases infection risk among night-shift workers (12–14).

The COVID-19 pandemic acted as a catalyst for the already existing trend in research on night-shift work and infection susceptibility. From the beginning of the pandemic, it was proposed that night-shift workers could be more susceptible to severe acute respiratory syndrome coronavirus-2 (SARS-CoV-2) (15–17), and that epidemiological studies were needed to provide more insight into this association. In the years that followed, multiple studies have responded to this request [for example (18–20)].

However, a clear overview summarizing the results of research into the association between night-shift work and susceptibility to SARS-CoV-2 infection and other infectious diseases is currently lacking. Such an overview is important considering the impact of infectious diseases on workers and society and could contribute to the development of strategies aimed at protecting night-shift workers from infection-related health risks. The aim of this systematic review and meta-analysis was therefore to synthesize the evidence in the scientific literature regarding the association between night-shift work and susceptibility to infectious diseases.

## Methods

To synthesize the scientific evidence on night-shift work and susceptibility to infectious diseases, we conducted a systematic review and meta-analysis using the Preferred Reporting Items for Systematic Reviews and Meta-Analyses (PRISMA) guidelines. The protocol is registered in the International Prospective Register of Systematic Reviews (PROSPERO) with registration number CRD42024594231.

### Eligibility criteria

To identify relevant studies, the following PECO [population, exposure, control, outcome (21)] outline was formulated: (i) *Population*: workers in all types of paid work aged ≥18 years; (ii) *Exposure*: night-shift work. Being exposed to work that is performed during the night-time hours. A broad definition is working ≥1 hour between 00:00–06:00 hours. This can be in a permanent night-shift work schedule (working predominantly or only night shifts), a rotating schedule (rotating between night shifts and morning, day, and/or evening shifts), and an irregular schedule (working night shifts, but not in a fixed pattern). Assessed by self-report and/or by registered data on work schedules; (iii) *Control*: non-shift work (workers who only work during the day). Not being exposed to work that is performed during the night-time hours, but working only during the day. Assessed by self-report and/or by registered data on work schedules; (iv) *Outcome*: infectious diseases. Including, but not limited to: common cold, influenza(-like illness), respiratory infections, SARS-CoV-2 infection, gastrointestinal infections, urinary tract infections, and Q-fever infection. Having acquired an infectious disease in a particular time frame (yes versus no), ie, incidence rate of infectious diseases. Assessed by self-report, test results and/or diagnosis by a physician. Studies must report at least one infectious disease outcome. In the case of SARS-CoV-2 infection, studies could potentially only describe the presence of the virus, without necessarily indicating the occurrence of COVID-19 disease.

Other inclusion criteria were that the studies were original studies with an observational design (ie, cross-sectional, retrospective, or prospective cohort studies, or case-control studies), that studies were published in a peer-reviewed journal from the start (no date restrictions) up to September 2024 in English, and that the full text of the study articles was available. Furthermore, studies were only included if they reported effect estimates and 95% confidence intervals (CI) or data to calculate these estimates of the association between night-shift work and infection susceptibility. Case reports, letters, reviews, preprint studies, and conference abstracts were excluded.

### Information sources and search strategy

The search strategy was formulated with the assistance of an experienced librarian. The bibliographic databases Embase and PsycINFO were searched from inception to 10 September 2024. The search strategy consisted of a component identifying the exposure (search terms: night shift worker, night shift, night work, nightshift, nightwork, overnight shift, overnight work, shift work, shift worker, working shift, rotating shift, rotating hours, irregular shift, irregular hours) and the outcome (search terms: infection, infectious, influenza, covid, corona, coronavirus disease 2019, sars-cov-2, q fever). The final search strategies in Embase and PsycINFO can be found in the supplementary material (www.sjweh.fi/article/4225), tables S1 and S2.

### Selection and data collection process

Two authors independently screened all records for inclusion. First, abstracts and titles were screened. Next, a screening of the full-text article was conducted for articles that were selected based on abstract and title. Subsequently, one author extracted data from the included studies, which was checked by a second author. Rayyan (www.rayyan.ai) was used during the screening and selection process of the articles to independently screen articles, to document the inclusion of articles, and to register why others were excluded.

### Data extraction items

The following data was extracted from the included studies: authors, year of publication, country, study design, population, number of participants, description of exposure and control (night-shift and non-shift work definition, and timing of assessment), description of outcome (infectious diseases definition, used measuring instruments, and timing of assessment), statistical analysis (methods and confounders), results (effect estimates and 95% CI adjusted for confounders), key conclusions of authors.

### Risk of bias assessment

The risk of bias in the included studies was assessed by two authors using the Newcastle-Ottawa Scale (NOS), a widely used tool for assessing the risk of bias in non-randomized studies (22). It consists of four items for selection of study groups, one item for comparability of the groups, and three for the ascertainment of the outcome. Every item can be scored 1 point, except for the comparability item that can be scored 2 points, resulting in a scale from 0–9 points. A score of 0–3, 4–6, and 7–9 indicates a high, moderate, and low risk of bias, respectively. For cross-sectional studies, an adapted version of the NOS was used [based on the version of Herzog et al (23)]. In this scale, two items are available for the ascertainment of the outcome, but one of these items can be scored 2 points instead of 1, resulting in a similar scale from 0–9. However, it should be noted that the NOS scores for cohort and cross-sectional studies cannot be directly compared because the items they assess differ. For the comparability item between night-shift and non-shift workers, occupation or another key work-related infection exposure variable was selected as the most important confounding factor. Sociodemographic factors (e.g. age and education), vaccination status, and infectious disease exposure at home – were selected as important additional confounding factors. The complete NOS tools used in this review are available in supplementary texts S1 and S2.

### Effect measures, synthesis methods, and reporting bias and certainty assessment

To provide a synthesized description of the results of the included studies, they were grouped based on the type of infection that was examined (ie, common respiratory infections (flu and common cold), SARS-CoV-2 infection, and other infections). To further synthesize the results, a meta-analysis was performed for common respiratory infections and SARS-CoV-2 infection. For the meta-analysis, we pooled the adjusted effect estimates. Effect estimates, including risk ratios (RR), odds ratios (OR), and hazard ratios (HR), and their 95% CI were used to calculate a pooled effect estimate. To this end, the effect estimates of the individual studies were transformed into log metrics. Next, these log metrics were included in random-effects meta-analyses with restricted maximum likelihood as estimation method, as has been previously recommended (24, 25). Random-effects models were chosen, because they assume that underlying true effects vary between studies, which is likely to be the case since population characteristics and exposure and outcome definitions generally differ across observational studies. The inverse variance method was applied to pool individual study effects. Overall effect estimates, indicated as OR, and 95% CI were reported and forest plots were constructed. To assess statistical heterogeneity, the I^2^ statistic was used, which quantifies the amount of variation between studies that cannot be attributed to chance. Based on the Cochrane Handbook cut-off points, an I^2^ of 0–40% can be considered low, 30–60% moderate, 50–90% substantial, and 75–100% high heterogeneity (26). When multiple studies included the same source population, only the study with lowest risk of bias or that aligned best with the review’s PECO outline was included to avoid double-counting data in the meta-analysis. Some studies reported multiple relevant effect estimates due to the inclusion of more than one group of night-shift workers or multiple infectious disease outcomes (ie, both flu and common cold). To assess the potential impact of correlation between results from the same study, a sensitivity analysis was conducted in which only one result per study was included, selecting the result based on the largest sample size. If the number of included studies permitted, subgroup analyses were conducted by stratifying analyses based on study design (prospective cohort study versus cross-sectional study), risk of bias (high versus moderate versus low), measurement of outcome (self-report versus serology/PCR tests), and occupational class (healthcare versus other occupational class). Meta-regression was used to test subgroup differences. Publication bias was assessed using a funnel plot, the Egger's test, and the trim-and-fill method. The Grading of Recommendations, Assessment, Development and Evaluations (GRADE) framework was used to assess the body of evidence (27).

## Results

### Study selection

**Figure 1:**
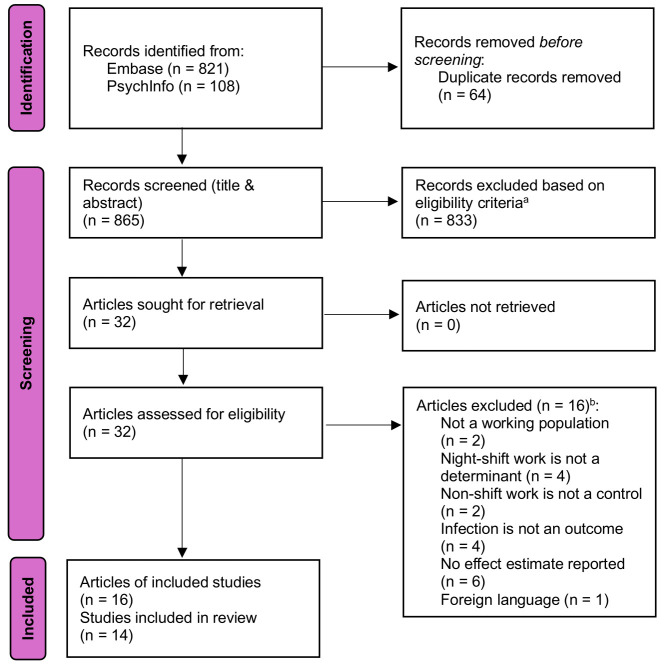
PRISMA 2020 flow diagram of included studies. ^a^ Reasons for exclusion were: not an original study, not a working population, night-shift work is not a determinant, non-shift work is not a control, infection is not an outcome, and qualitative study. ^b^ Three articles were excluded for two reasons. Therefore, the numbers per reason add up to 19 instead of 16.

Figure 1 shows a flow diagram of the identification and inclusion of studies. In total, 929 records were identified from Embase and PsycINFO. After removing duplicates, the titles and abstracts of 865 records were screened for potential inclusion. Next, the full-texts of 32 articles meeting the eligibility criteria were retrieved and assessed for eligibility. From this selection, 16 articles were excluded because they did not meet the inclusion criteria for the following reasons: the study population was not a working population (28, 29), night-shift work was not a determinant (30–33), non-shift work was not a control (34, 35), infection was not an outcome (34, 36–38), no effect estimate was reported (28, 31, 39–42), and the article was not published in English (43). Hence, 16 articles covering 14 studies were included in this review (18–20, 44–54). The studies of Fatima et al (19) and Maidstone et al (55) both included participants from the UK Biobank population, and the studies of Coppeta et al (18) and Rizza et al (56) included participants from the same Italian hospital, and therefore only the two studies with the longest follow-up periods were included in the results synthesis of the current review.

### Study characteristics

Of the 14 included studies, 4 described prospective cohort studies (18, 19, 46, 47) and 10 described cross-sectional studies (20, 44, 45, 48–54) (table 1). Studies were conducted in Europe (N=8) (18, 19, 44–49), North America (N=3) (50–52), Asia (N=2) (53, 54), and South America (N=1) (20). Only 3 studies were published before 2020 (47, 49, 53). Workers from various occupational classes were assessed in 7 studies (19, 44–46, 49–51), 5 studies included healthcare workers (18, 20, 47, 48, 52), 1 study included factory workers (53) and another university employees (54). In 9 studies, an overall night-shift worker group was compared to a non-shift worker group (18, 20, 44–47, 52–54), while, in the other 5 studies, night-shift workers were categorized in different subgroup based on night-shift work schedule or frequency (eg, sometimes or usually/always night-shift work) (19, 48–51). In total, the studies included 191 320 participants, with the number of participants per study varying between 336 and 85 529 participants. Of the included participants, 25% were night-shift workers and 75% were non-shift workers. Most studies solely focused on SARS-CoV-2 as outcome (N=9) (18–20, 44, 46, 48, 51, 52, 54). The other 5 studies focused on hepatitis A (53), respiratory infections (47), head or chest cold (50), common infections (common cold, flu-like illness, and gastroenteritis) (49), and various infections (common cold, throat infection, ear infection, sinusitis, pneumonia/bronchitis, SARS-CoV-2, influenza-like illness, skin infection, gastrointestinal infection, urinary infection, venereal disease, and eye infection) (45).

**Table 1:** Characteristics of included studies. [CSS=cross-sectional study; inf=infection; PCS=prospective cohort study]

Study	Country	Design	Population	Sample	Data collection	Exposure and control	Outcome (assessment)
Bjorvatn et al 2023 (44)	Multi-national	CSS	Working adults from the International COVID Sleep Study	6076	May–Dec 2021	1. Shift/night work 2. Regular day work	SARS-CoV-2 (self-report)
Bjorvatn et al 2024 (45)	Norway	CSS	Working adults recruited through their general practitioner via the Norwegian practice-based research network in general practice (PraksisNett).	867	Mar 2022– Jan 2023	1. Shift work including night work 2. Non-shift work	Common cold, throat infection, ear inf, sinusitis, pneumonia-bronchitis, SARS-CoV-2, influenza-like illness, skin inf, gastrointestinal inf, urinary inf, venereal disease, eye inf (self-report)
Coppeta et al 2021 (18)	Italy	PCS	Nurses working in an Italian hospital	916	Mar–Dec 2020	1. Night-shift work (2–7 nights/month) 2. Daytime work (never work at night)	SARS-CoV-2 (serology/PCR)
Fatima et al 2021 (19)	United Kingdom	PCS	Working adults from the UK Biobank who were tested for COVID-19 infection	8394	2006–2010 and Mar–Sep 2020	1. Mixed-shift work (sometimes/usually/always working a shift but only sometimes working a night shift) 2. Night-shift work (sometimes/usually/always working a shift and usually/always working a night shift) 3. Non-shift work (not working shifts at all)	SARS-CoV-2 (PCR)
Loef et al 2019 (47)	Nether-lands	PCS	Healthcare workers from 6 different hospitals in the Netherlands (from the Klokwerk+ Study)	589	Sep 2016– June 2017	1. Shift work (rotating and/or night shifts; all shift workers worked rotating shifts, and 93% of shift workers also worked night shifts) 2. Non-shift work (not working rotating shifts or night shifts; working day shifts only)	Respiratory infections (self-report)
Loef et al 2022 (46)	Nether-lands	PCS	Working adults from the Dutch population-based Lifelines COVID-19 cohort	26 051	Mar 2020– Mar 2021	1. Night-shift work (regularly or occasionally) 2. Day work (not working night shifts)	SARS-CoV-2 (self-report)
Martin et al 2022 (48)	United Kingdom	CSS	Healthcare or ancillary workers in a healthcare setting and/or registered with a UK professional regulatory body in healthcare	10 656	Dec 2020– Mar 2021	1. Working nights less than weekly 2. Working nights weekly or always 3. Never working nights	SARS-CoV-2 (self-report)
Mohren et al 2002 (49)	Nether-lands	CSS	Large heterogeneous population of employees working in 45 different companies and organizations from the Maastricht Cohort Study	8255	May 1998	1. Three-shift work (involves three alternating teams, with a semi-continuous schedule) 2. Five-shift work (full continuous shift work, including five alternating teams) 3. Irregular shift work (involves frequently deviating work hours, but includes frequent night work) All shift work schedules include night work 4. Day work (working daytime only)	Common cold, flu-like illness, gastroenteritis (self-report)
Prather et al 2021 (50)	United States	CSS	Working adults from the National Health Interview Survey (nationally representative sample)	33 407	2010 or 2015	1. Regular night-shift schedule 2. Rotating shift schedule 3. Regular daytime schedule	Head or chest cold (self-report)
Quan et al 2024 (51)	United States	CSS	Working adults participating in public health surveillance surveys in the United States	8732	Mar–Aug 2022	1. Day and/or evening shifts and some night shifts 2. Night shift only Night shift is any shift in which the majority of the work hours occur between 22:00–08:00 hours. 3. Day shift only (occurs any time between 06:00–19:00 hours	SARS-CoV-2 (self-report)
Swanson et al 2023 (52)	United States	CSS	Nurses working in a hospital or out-patient setting (invited through the American Nursing Association)	336	May 2020– Apr 2021	1. Night-shift work (nights shifts are from 19:00–07:00 hours) 2. Standard shift work (standard shifts are from 08:00–17:00 hours)	SARS-CoV-2 (self-report)
Wang et al 1990 (53)	China	CSS	Factory workers from the Shanghai No 2 Yarn Dyeing and Weaving Mill	899	Apr 1988	1. Day/night shift (in one week, two days f rom 07:00–15:00 hours, the following two days from 15:00–23:00 hours and last two days from 23:00–07:00 hours) 2. Day shift	Hepatitis A (serology)
Widyahening et al 2022 (54)	Indonesia	CSS	University administrative employees and security personnel from Universitas Indonesia	613	Oct 2021	1. Shift work (including night shifts) 2. Non-shift work	SARS-CoV-2 (self-report)
Zuñiga et al 2022 (20)	Chile	CSS	Healthcare workers in the Chilean public health care system (nationwide study)	85 529	Sep–Oct 2020	1. Night-shift work (8–12 hour weekdays with one 12-hour night shift, 12-hour day shift- 12 hours night shift-2 days off, or 24 hour shift-3 days off) 2. Day shifts (8-12 hour day shift)	SARS-CoV-2 (serology/self-report)

### Risk of bias

Table 2 indicates for each study the risk of bias in each domain (selection, comparability, and outcome) and an overall study-level risk of bias. All prospective cohort studies scored 5–6 stars on the NOS, indicating a moderate risk of bias in these studies. While almost all prospective cohort studies scored well on representativeness of the night-shift worker cohort and selection of the non-shift worker cohort, the ascertainment of the exposure was based on self-report in all studies, and only one study described the adequacy of the follow-up of cohorts. The results of the risk of bias assessment for the cross-sectional studies were more diverse, with 2 studies scoring 3 stars (high risk of bias), 3 studies scoring 6 stars (moderate risk of bias), and 5 studies scoring 7–8 stars (low risk of bias). All cross-sectional studies had an adequate sample size and used appropriate statistical tests. However, nearly all cross-sectional had unsatisfactory response rates (<50%) and/or did not describe the characteristics of the responders and the non-responders to assess the comparability between these groups. In only 8 of the 14 included studies, the analyses were adjusted for occupation or another important work-related infection exposure variable. In contrast, nearly all studies did adjust for age as a relevant sociodemographic factor.

**Table 2:** Risk of bias assessment of included studies with the Newcastle-Ottawa Scale (NOS). [CSS=cross-sectional study; PCS=prospective cohort study]

Study	Study design	Selection (4 stars)	Comparability (2 stars)	Outcome (3 stars)	Total ^a^ (9 stars)
Coppeta et al (18)	PCS	1	2	2	5 (moderate)
Fatima et al (19)	PCS	3	1	2	6 (moderate)
Loef et al (47)	PCS	2	2	2	6 (moderate)
Loef et al (46)	PCS	2	2	1	5 (moderate)
Bjorvatn et al (44)	CSS	3	2	2	7 (low)
Bjorvatn et al (45)	CSS	3	1	2	6 (moderate)
Martin et al (48)	CSS	2	2	2	6 (moderate)
Mohren et al (49)	CSS	3	2	2	7 (low)
Prather et al (50)	CSS	3	1	2	6 (moderate)
Quan et al (51)	CSS	3	2	2	7 (low)
Swanson et al (52)	CSS	2	0	1	3 (high)
Wang et al (53)	CSS	3	1	3	7 (low)
Widyahening et al (54)	CSS	1	0	2	3 (high)
Zuñiga et al (20)	CSS	3	2	3	8 (low)

^a^ A NOS score of 0–3, 4–6, and 7–9 indicates a high, moderate, and low risk of bias, respectively.

### Study results

Supplementary table S3 presents the statistical analysis, the included confounders, and the results (ie, effect estimates and 95% CI) of the included studies. These adjusted effect estimates were included in the meta-analysis. In the next paragraphs, the results are discussed per type of infection that was examined: common respiratory infections (flu and common cold), SARS-CoV-2 infection, and other infections.

### Common respiratory infections

**Figure 2:**
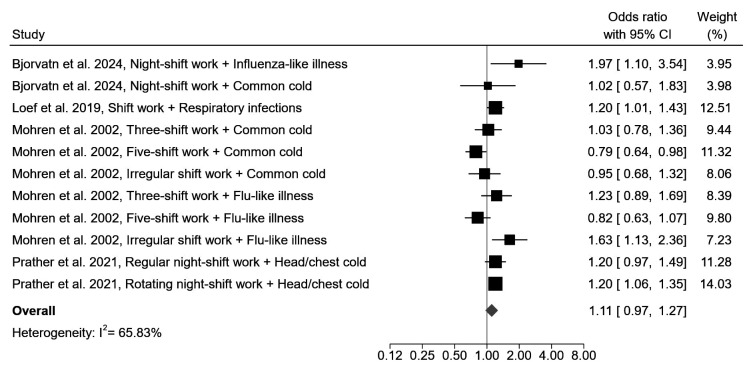
Forest plot of the association between night-shift work and common respiratory infections.[CI=confidence interval.]

In total, 4 studies (1 prospective and 3 cross-sectional), with a low-to-moderate risk of bias, examined the association between night-shift work and common respiratory infections. These were (common) cold (45, 49, 50), influenza-like illness (45, 49), or respiratory infections (both common cold and influenza-like illness) (47). Prather et al (50) reported an increased risk of head or chest cold among rotating night-shift workers (OR 1.20, 95% CI 1.06–1.35), but Mohren et al (49) and Bjorvatn et al (45) did not find an increased risk of common cold among night-shift workers. For five-shift workers (full continuous shift work, including five alternating teams), Mohren et al (49) even found a reduced risk of common cold compared to day workers (OR 0.79, 95% 0.64–0.98), though irregular shift workers (involves frequently deviating work hours, but includes frequent night work) were more likely to have influenza-like illness than day workers (OR 1.63, 95% CI 1.13–2.36). In correspondence, Bjorvatn et al (45) found an association between night-shift work and influenza-like illness (OR 1.97, 95% CI 1.10–3.55). The study of Loef et al (47), which was the only prospective cohort study as well as the only study among healthcare workers, concluded that shift workers had a 20% higher incidence of respiratory infections than non-shift workers (IRR 1.20, 95% CI 1.01–1.43). Taken together, the results of the meta-analysis did not reveal a statistically significantly increased risk of common respiratory infections among night-shift compared to non-shift workers (OR 1.11, 95% CI 0.97–1.27, I^2^=65.8%) (figure 2). In the sensitivity analysis in which only one effect estimate per study was selected, this was OR was 1.05 (95% CI 0.84–1.32, I^2^=78.2%) (supplementary figure S1).

### SARS-CoV-2 infection

**Figure 3:**
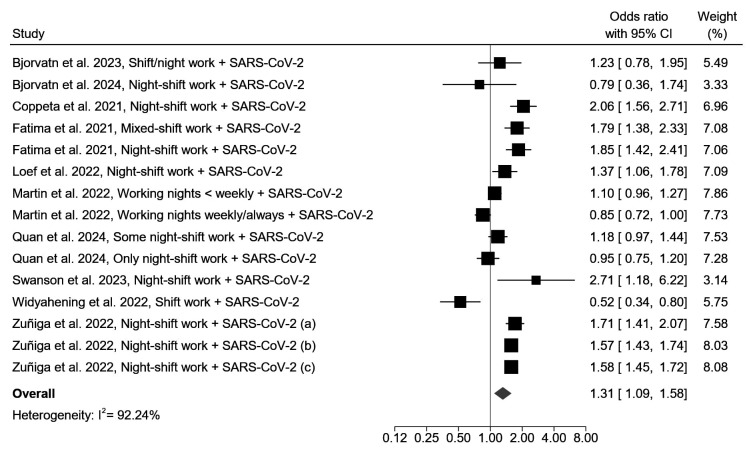
Forest plot of the association between night-shift work and SARS-CoV-2 infection. In the study of Zuñiga et al (20), due to significant variations in the rate of seropositivity in the various regions of Chile, regions were grouped into three categories: a) regions with <4% seropositivity, b) regions with 4-8% seropositivity, and c) regions with >10% seropositivity. [CI=confidence interval.]

The association between night-shift work and SARS-CoV-2 infection was examined in 10 studies (3 prospective and 7 cross-sectional). Of these, 5 reported an increased risk of SARS-CoV-2 infection among night-shift workers (18–20, 46, 52), 4 reported no association between night-shift work and SARS-CoV-2 infection (44, 45, 48, 51), and 1 reported a decreased risk of SARS-CoV-2 infection among night-shift workers (54). When meta-analyzing the results of these studies, night-shift workers had a higher risk of SARS-CoV-2 infection than non-shift workers (OR 1.31, 95% CI 1.09–1.58, I^2^=92.2%) (figure 3, supplementary table S4). The same result was observed when only one effect estimate per study was selected (OR 1.31, 95% CI 1.02–1.69, I^2^=90.7%) (supplementary figure S2).

The association between night-shift work and SARS-CoV-2 infection was stronger in the 3 prospective cohort studies (OR 1.74, 95% CI 1.47–2.06, I^2^=38.3%) than the 7 cross-sectional studies (OR 1.17, 95% CI 0.94–1.46, I^2^=93.2%) (supplementary figure S3, table S4). Furthermore, when stratifying the results based on risk of bias, it was observed that the meta-analyzed results of the 5 studies with a moderate risk of bias (OR 1.36, 95% CI 1.04–1.78, I^2^=88.8%) and the 3 studies with a low risk of bias (OR 1.37, 95% CI 1.14–1.65, I^2^=87.1%) showed a statistically significant association between night-shift work and SARS-CoV-2 infection, while this was not observed for the 2 studies with a high risk of bias (OR 1.14, 95% CI 0.23–5.74, I^2^=91.7%) (supplementary figure S4, table S4). In the study of Widyahening et al (54), the only study that found a decreased risk of SARS-CoV-2 infection in night-shift workers, all non-shift workers were administrative employees while all night-shift workers were security personnel who worked individually and mostly outdoors. As the analysis was not adjusted for occupation or other important work-related infection exposure variables, the risk of bias in this study was high.

Interestingly, the studies that did find an increased risk of SARS-CoV-2 infection among night-shift workers were generally conducted in the first year of the COVID-19 pandemic, while the studies that did not observe this result were conducted later. A subgroup meta-analysis indeed showed that the increased OR of SARS-CoV-2 infection was 1.51 (95% CI 1.26–1.81, I^2^=90.8%) in the 6 studies conducted before April 2021 (first year of the pandemic) and 0.92 (95% CI 0.68–1.26, I^2^=74.4%) in the 4 studies conducted from April 2021 onwards (supplementary figure S5 and table S4). The association between night-shift work and SARS-CoV-2 infection was also stronger in studies that measured SARS-CoV-2 using serology or PCR tests (OR 1.63, 95% CI 1.54–1.72, I^2^=0%, 3 studies) versus self-report (OR 1.04, 95% CI 0.85–1.27, I^2^=79.9%, 7 studies), and in studies among healthcare workers (OR 1.46, 95% CI 1.14–1.88, I^2^=94.5%, 4 studies) versus various occupational classes (OR 1.17, 95% CI 0.88–1.56, I^2^=85.9%, 6 studies), though the latter subgroup difference was not statistically significant (supplementary figure S6 and S7, table S4).

### Other infections

Besides common respiratory infections and SARS-CoV-2 infection, a few other types of infections have been studied in relation to night-shift work in 3 cross-sectional studies. Wang et al (53) studied the association between night-shift work and hepatitis A infection during an epidemic in a factory in Shanghai. Even after adjusting for the major source of infection (ie, eating clams), night-shift work was an independent risk factor for hepatitis A (OR 2.55, 95% CI 1.40–4.63) in this study with a low risk of bias. Mohren et al (49) found an increased risk of gastrointestinal infections among five-shift workers (OR 1.42, 95% CI 1.05–1.91). However, this was not observed among three-shift workers and irregular shift workers and also not among night-shift workers from the study of Bjorvatn et al (45) (OR 1.29, 95% CI 0.66–2.52). For several other infection types, Bjorvatn et al (45) also did not observe an association with night-shift work, these were throat infection, ear infection, sinusitis, pneumonia/bronchitis, skin infection, urinary infection, venereal disease, and eye infection. Since only 1–2 studies were conducted for the different other types of infections, no meta-analysis was performed.

### Publication bias assessment

Visual inspection of the funnel plots did not reveal a clear indication of publication bias (supplementary figure S8). This was supported by the regression-based Egger test for small-study effects that yielded a non-significant intercept (P=0.3426 for common respiratory infections and P=0.7611 for SARS-CoV-2) and the trim-and-fill method that did not impute additional hypothetical studies in the funnel plots.

### Certainty of evidence

Using the GRADE criteria, the level of evidence of an association of night-shift work with common respiratory infections and with SARS-CoV-2 infection was graded very low (supplementary table S5). Because the evidence in the current review is based on observational studies, the initial level of evidence was already low, but this was further downgraded because of the possible risk of bias due to limitations in the design of the included studies and because of the high inconsistency in the studies as reflected by the I^2^ statistic.

## Discussion

In this systematic review, 14 studies with 191 320 participants were included that examined the association of night-shift work with common respiratory infections, SARS-CoV-2 infection, and other infectious diseases. The meta-analysis showed that night-shift work was associated with an increased risk of SARS-CoV-2 infection (OR 1.31, 95% CI 1.09–1.58, I^2^=92.2%) but not common respiratory infections (OR 1.11, 95% CI 0.97–1.27, I^2^=65.8%). Due to the scarcity of studies, no meta-analysis was performed for other infections. The certainty of evidence was graded very low due to a limited number of (prospective cohort) studies for each outcome and due to high inconsistency in the available studies.

### SARS-CoV-2 infection

The finding on the increased SARS-CoV-2 infection risk among night-shift workers is in line with a previous systematic review and meta-analysis that aimed to study the association between sleep disturbances and COVID-19 (57). In this previous meta-analysis, night-shift work was found to be associated with SARS-CoV-2 infection risk (OR 1.49, 95% CI 1.13–1.96). Zhou et al (57) also found an association between other sleep disturbances and SARS-CoV-2 infection, suggesting a key role for disturbed sleep in night-shift workers’ increased infection susceptibility. Similarly, the effect of disturbed sleep has been discussed in multiple reviews, describing that it causes alterations in the adaptive and innate immune system such as in the production of cytokines, the functioning of T cells and natural killer cells, and the humoral response (17, 58–60). In correspondence, disturbances in the number of white blood cell counts have been observed among night-shift workers (13) and in circadian rhythms of immune functions such as cytokine release after simulated night-shift work (14, 61). Disturbed sleep in combination with altered circadian rhythms of different bodily processes could also increase vulnerability to biological agents such as infectious pathogens during the night shift (17, 62). In other words, exposure to a pathogen during the night shift may carry a higher risk of infection than encountering that same pathogen during daytime hours, when the immune system is generally better prepared to combat pathogens (17, 62, 63). As a result, disturbed sleep and circadian rhythm disruption may enhance SARS-CoV-2 infection risk among night-shift workers.

### Common respiratory infections and other infections

Following this line of reasoning, a similar increased susceptibility to common respiratory infections among night-shift workers could be expected. For example, previous work has indicated that shorter sleep duration is associated with increased susceptibility to common cold (64, 65). However, our meta-analysis does not provide compelling evidence for an association between night-shift work and common respiratory infections, with an OR of 1.11 (95% CI 0.97–1.27). Nevertheless, since only 4 studies were identified that studied common respiratory infections as an outcome, more studies are needed to draw a more definitive conclusion. In addition, the included studies all relied on self-report instead of objective testing to assess the incidence or prevalence of common respiratory infections. Especially in studies with a cross-sectional design, which was the design of 3 of the 4 studies, recall bias regarding infection (symptoms) may have influenced the findings. Besides SARS-CoV-2 infection and common respiratory infections, several other infectious disease outcomes were studied in relation to night-shift work. For hepatitis A and gastrointestinal infection, some evidence was found for an positive association with night-shift work. Because a scarcity of studies was available for these outcomes, there is insufficient evidence to draw reliable conclusions.

### SARS-CoV-2 infection: Impact of subgroup analyses

The association between night-shift work and SARS-CoV-2 infection was stronger in prospective cohort versus cross-sectional studies (OR 1.74 versus 1.17), in studies with low-to-moderate risk of bias versus studies with high risk of bias (OR 1.37/1.36 versus 1.14), and in studies using serology/PCR tests versus studies using self-report (OR 1.63 versus 1.04). The higher-quality studies therefore provide greater support for this association compared to the lower-quality studies. The association between night-shift work and SARS-CoV-2 infection was also more pronounced among healthcare than other sectors (OR 1.46 versus 1.17), though this difference was not statistically significant in the meta-regression (OR 1.25, 95% CI 0.86–1.83). This finding might be related to the high exposure of SARS-CoV-2 in the work environment of (essential) healthcare workers. Previous studies have shown that healthcare workers were at greater risk for SARS-CoV-2 infection than other workers (66, 67). As a result, night-shift workers in healthcare may have faced a disproportionally higher exposure to SARS-CoV-2 than those in other sectors. However, another possible explanation is that all 4 studies among healthcare workers were conducted in the first year of the COVID-19 pandemic. When stratifying the results based on the data collection period, a strong association between night-shift work and SARS-CoV-2 infection was observed in studies conducted in the first year of the pandemic, while no such association was observed in studies conducted after this first year (OR 1.51 versus 0.92). Several factors could explain this observation. First, SARS-CoV-2 emerged as a novel virus, against which no pre-existing immunity was present in the population. Over time, more and more people experienced a SARS-CoV-2 infection leading to some level of natural immunity (68). Therefore, increasing population immunity and differences in the association with primary infection versus reinfection might have contributed to the diminishing association between night-shift work and SARS-CoV-2 infection after the first year of the pandemic. Furthermore, different variants of the virus occurred over time (69). Later SARS-CoV-2 variants with higher transmissibility but causing less severe disease may have resulted in a higher number of asymptomatic infections. This might have caused more misclassification of SARS-CoV-2 cases and weakened the association between night-shift work and SARS-CoV-2 infection. Second, after the first year of the pandemic, vaccines became available that not only prevented severe COVID-19, but also prevented transmission of SARS-CoV-2 (70, 71). In some studies, vaccine uptake was found to be higher in night-shift workers than non-shift workers (44, 46), which could weaken the association with SARS-CoV-2 infection. The higher vaccine uptake in these studies may be attributed to the fact that night-shift workers were more often in essential occupations (eg, healthcare workers) and generally had earlier and more access to vaccines. On the other hand, vaccination success may be reduced among night-shift compared to non-shift workers due to disturbed sleep and circadian rhythm disruption (72). This would result in a stronger association after the introduction of SARS-CoV-2 vaccines. However, a recent study found night-shift work to have little-to-no effect on the primary immune response to SARS-CoV-2 vaccination (73). Lastly, testing advice and accessibility changed during the course of the pandemic, which influenced overall test uptake (74). After the first year of the pandemic, antigen self-tests became widely available. While some studies made use of serology and/or PCR tests to assess SARS-CoV-2 infection, most studies, and all of those conducted after the first year of the pandemic, relied on self-report. Therefore, differences in self-testing behavior may have influenced the results particularly after the first year of the pandemic.

### Strengths and limitations

A strength of this first review into the association between night-shift work and infection susceptibility is that it applied a systematic approach to include relevant studies, assess their risk of bias, summarize the results, and appraise the certainty of evidence using GRADE. Despite this approach, it should be noted that risk of bias assessment tools such as the NOS, while widely recognized, do not fully capture all methodological nuances or context-specific factors that could influence the quality of the included studies. However, the use of these tools contributes to ensuring consistency and comparability with regards to assessing risk of bias in all studies. All studies included in the meta-analysis reported OR, except for one study on SARS-CoV-2 that reported a HR, and one study on common respiratory infections that reported an IRR. A note of caution is due here since these measures are not identical to OR. As similar results were obtained when excluding these studies, we assume the impact of the different measures on our results to be limited.

The certainty of evidence in the meta-analysis was graded as very low. This was partly caused by the high inconsistency in the available studies. This heterogeneity may stem from several factors, including differences in study design, population characteristics, and the definition and assessment of the outcome between the studies. While high heterogeneity remained notable in the subgroup analyses for risk of bias, time, and occupational class of the population, heterogeneity was low in prospective cohort studies and studies using serology/PCR tests instead of self-report to measure SARS-CoV-2 infection. In most studies, SARS-CoV-2 infection was based on participants reporting positive tests results, indicating the presence of the virus. However, it is important to note that this does not provide direct information about the severity of the infectious disease.

Another source of heterogeneity may stem from the definition and assessment of night-shift work. A commonly used definition of night-shift work is working ≥3 hours between 23:00–06:00 hours (75, 76). In this review, we referred to a broader definition of working ≥1 hour between 00:00–06:00 hours, to ensure the inclusion of all potentially relevant studies. However, most of the included studies did not provide a clear definition of night-shift work and/or a description of the night-shift work schedule of the study population. Therefore, substantial differences in the shift system (eg, number of hours per day and rotating versus permanent schedule), shift intensity (eg, frequency of night shifts and time off between shifts), and duration (eg, years of night-shift work) may exist between studies. These are the major shift domains to be captured in epidemiological studies as formulated in the International Agency for Research on Cancer (IARC) working group report (77). Future original studies should ideally consider these different domains rather than relying solely on a dichotomous measure of working night shifts or not to obtain a more comprehensive insight into night-shift work exposure. Subsequently, when such studies are synthesized, stratified analyses could be performed to examine factors such as rotating versus permanent night-shift work, and frequency and duration of night-shift work.

Half of the included studies failed to adjust for occupation or other important work-related infection exposure variables. Such adjustments are important, because night-shift and non-shift workers are likely to differ in work characteristics other than working night shifts that may affect their susceptibility to infections and their access to diagnostic testing. For example, in some countries, SARS-CoV-2 testing was linked to occupation, with compulsory testing for healthcare workers and other essential workers. These occupational differences in infection susceptibility and testing access could introduce bias when night-shift workers are overrepresented in high-risk occupations. While studies conducted within a single occupation or even a single organization have the advantage that confounding due to differences between night-shift and non-shift workers is reduced, the generalizability of these studies to the general population is often limited (7). Therefore, population-based studies are needed that adequately take into account work-related confounding factors. At the same time, studies should be cautious about adjusting for factors that potentially lie in the causal pathway between night-shift work and infection susceptibility. For example, in multiple studies, associations were adjusted for sleep (19, 49, 51). Considering the possible underlying role of sleep in the association between night-shift work and infection susceptibility, adjustment for sleep could be considered undesirable as it may lead to an underestimation of the effect being studied.

### Concluding remarks

In conclusion, the findings of this systematic review and meta-analysis suggest that night-shift work is associated with increased risk of SARS-CoV-2 infection (OR 1.31, 95% CI 1.09–1.58, I^2^=92.2%) but not common respiratory infections (OR 1.11, 95% CI 0.97–1.27, I^2^=65.8%). However, the certainty of evidence for these associations is very low due to a limited number of available studies with substantial heterogeneity between them. Therefore, more research is needed on the association between night-shift work and different infection diseases outcomes. In particular, future studies are recommended that apply a prospective design with appropriate adjustment for confounding factors, collect extensive information on night-shift work exposure, and ideally use objective methods to assess incidence of infectious diseases.

## Supplementary material

Supplementary material
