# Supplementary material for: Night-shift work and susceptibility to infectious diseases: a systematic review and meta-analysis
Source: Scand J Work Environ Health. 2025 Jun 26;51(4):298–311. doi: 10.5271/sjweh.4225 (PMC12281634; doi:10.5271/sjweh.4225)
Supplement: Supplementary material [file SJWEH-51-298-S001.pdf]

Night-shift work and susceptibility to infectious diseases: a systematic review and meta-analysis[1]

by Bette Loef, PhD,[2] Esmee Bosma, MSc, Linda W M van Kerkhof, PhD, Karin I Proper, PhD, Debbie van Baarle, PhD, Martijn E T Dollé, PhD

1. Supplementary material
2. Correspondence to: Bette Loef, PhD, Center for Prevention, Lifestyle and Health; National Institute for Public Health and the Environment; P.O. Box 1, 3720 BA, Bilthoven, The Netherlands. [E-mail: bette.loef@rivm.nl]

Table S1. Search strategy Embase (search performed September 10, 2024)

Rule	Query	Results
#1	'night shift worker'/exp OR ('shift*':ti AND 'night*':ti)	2316
#2	'night shift'/exp OR 'night shift*':ti,ab OR 'night work':ti,ab OR 'nightshift*':ti,ab OR 'nightwork*':ti,ab OR 'overnight shift*':ti,ab OR 'overnight work*':ti,ab OR 'rotating shift*':ti,ab OR 'rotating hours*':ti,ab OR 'irregular shift*':ti,ab OR 'irregular hours*':ti,ab	9799
#3	'shift work'/exp OR 'shift worker'/exp OR 'shift work*':ti,ab OR 'working shift*':ti	16255
#4	#1 OR #2 OR #3	18922
#5	'infection'/exp/mj OR 'infectious*':ti OR 'infection*':ti OR 'covid*':ti OR ('corona*':ti NOT ('coronair*':ti OR 'coronary*':ti)) OR 'q fever*':ti OR 'influenza'/exp OR 'influenz*':ti OR 'coronavirus disease 2019'/exp OR 'sars-cov-2*':ti OR 'sars-cov2*':ti	3407542
#6	#4 AND #5	821

Table S2. Search strategy PsycInfo (search performed September 10, 2024)

Rule	Query	Results
#1	exp workday shifts/	2789
#2	("Night shift*" or "Nightshift*" or "Shift work*" or "Working shift*" or "Nightwork*" or "Overnight shift*" or "Overnight work*" or "Rotating shift*" or "Rotating hours*" or "Irregular shift*" or "Irregular hours*").ti,ab.	3241
#3	1 or 2	4375
#4	exp Infectious Disorders/	117056
#5	exp Influenza/	1777
#6	exp COVID-19/	39441
#7	("Infection*" or "infectious*" or "covid*" or "corona" or "q-fever*" or "influenz*" or "sars-cov2*" or "sars-cov 2").ti,ab.	100100
#8	4 or 5 or 6 or 7	148395
#9	3 and 8	108

Table S3. Results of included studies. [CI: confidence interval; HR: hazard ratio; IRR: incidence rate ratio; OR: odds ratio]

Study	Statistical analysis	Confounders	Results
Bjorvatn et al. 2023 (44)	Logistic regression, weighted using the population age and sex distribution of each country	Age, age^2, sex, marital status, highest attained education, ethnicity, children living at home, obesity, vaccination status, and face-to-face work	Shift/night work -> SARS-CoV-2: OR=1.23 (95%-CI 0.78-1.96)
Bjorvatn et al. 2024 (45)	Logistic regression	Age, gender, marital status, country of birth, children living at home, and educational level	Shift work including nights was associated with higher odds of influenza-like illness (OR=1.97, 95%-CI 1.10–3.55), but none of the other infections. Common cold: OR=1.02 (95%-CI 0.57-1.83), throat infection: OR=1.41 (95%-CI 0.67-2.99), ear infection: OR=0.67 (95%-CI 0.15-3.09), sinusitis: OR=1.39 (95%-CI 0.58-3.30), pneumonia/bronchitis: OR=2.18 (OR=0.71-6.72), COVID-19: OR=0.79 (95%-CI 0.36-1.75), skin infection: OR=0.75 (95%-CI 0.35-1.60), gastrointestinal infection: OR=1.29 (95%-CI 0.66-2.52), urinary infection: OR=1.25 (95%-CI 0.35-4.49), venereal disease: OR=0.56 (OR=0.07-4.66), eye infection: OR=1.42 (95%-CI 0.56-3.57)
Coppeta et al. 2021 (18)	Logistic regression	Age, BMI, sex, smoking status, working in high risk settings	Night-shift work -> SARS-CoV-2: OR=2.06 (95%-CI 1.32-2.30)
Fatima et al. 2021 (19)	Logistic regression	Age, gender, ethnic minority status, income, education, Townsend deprivation quintile, sleep variables (sleep duration, daytime sleepiness, sleeplessness, snoring), obesity, overall health	Mixed-shift work -> SARS-CoV-2: OR=1.79 (95%-CI 1.38-2.33)<br>Night-shift work -> SARS-CoV-2: OR=1.85 (95%-CI 1.42-2.41)
Loef et al. 2019 (47)	Negative binomial regression, with the number of completed diaries as offset variable	Age, sex, occupation, influenza vaccination status, and general perceived health	Shift work -> respiratory infections: IRR=1.20 (95%-CI 1.01-1.43)
Loef et al. 2022 (46)	Cox proportional hazards regression	Age, sex, educational level, household composition, occupation, occupational class, working (partly) from home, occupation involving frequent contact with others, BMI, and smoking	Night-shift work -> SARS-CoV-2: HR=1.37 (95%-CI 1.05-1.77)
Martin et al. 2022 (48)	Logistic regression	Age, sex, ethnicity, migration status, religiosity, index of multiple deprivation, household size, cohabitation, accommodation, social mixing with others outside household, comorbidities, shielding status, smoking status, COVID-19 vaccination status, region of workplace, time between questionnaire rollout and completion, occupation, transport to work, number of SARS-CoV-2-positive patients attended to per week (with physical contact), access to appropriate	Working nights less than weekly -> SARS-CoV-2: OR=1.10 (95%-CI 0.96-1.27)<br>Working nights weekly or always -> SARS-CoV-2: OR=0.85 (95%-CI 0.72-1.00)

		personal protective equipment, aerosol generating procedure exposure, work areas	
Mohren et al. 2002 (49)	Multilevel logistic regression, in which individual employees were nested within job titles	Age, gender, longstanding disease, smoking, drinking habits, exercise, psychological job demands, decision latitude, physical demands, emotional demands, sleep quality, fatigue	Common cold: 3-shift work: OR=1.03 (95%-CI 0.78-1.36), 5-shift work: 0.79 (0.64-0.98), irregular shift work: 0.95 (0.68-1.32).<br>Flu-like illness: 3-shift work: OR=1.23 (95%-CI 0.90-1.70), 5-shift work: 0.82 (0.63-1.07), irregular shift work: 1.63 (1.13-2.36).<br>Gastroenteritis: 3-shift work: OR=1.06 (95%-CI 0.73-1.54), 5-shift work: 1.42 (1.05-1.91), irregular shift work: 1.17 (0.73-1.88).
Prather et al. 2021 (50)	Logistic regression	Age, gender, race, educational attainment, income, marital status, smoking status, alcohol consumption, physical activity, body mass index, presence of medical comorbidities, and survey year	Regular night-shift schedule -> head or chest cold: OR=1.20 (95%-CI 0.97-1.49)<br>Rotating shift schedule -> head or chest cold: OR=1.20 (95%-CI 1.06-1.35)
Quan et al. 2024 (51)	Logistic regression	Age, sex, race, BMI, vaccination status (boosted vs not boosted), # of the following conditions: diabetes, asthma, sickle cell disease, cardiovascular disease, hypertension, cancer, chronic kidney disease, liver disease, and chronic obstructive pulmonary disease, and the following factors: education, income, employment, sleep duration, percent time working remotely, and employment sector.	Day/evening and some night shifts -> SARS-CoV-2: OR=1.18 (95%-CI 0.97-1.44)<br>Night shifts only -> SARS-CoV-2: OR=0.95 (95%-CI 0.75-1.20)
Swanson et al. 2023 (52)	Logistic regression	Alcohol subtype	Night-shift work -> SARS-CoV-2: OR=2.71 (95%-CI 1.18-6.21)
Wang et al. 1990 (53)	Logistic regression	Age, eating clams, eating out	Day/night shift -> hepatitis A: OR=2.55 (95%-CI 1.40-4.63)
Widyahening et al. 2022 (54)	Logistic regression	Smoking, BMI, waist circumference, systolic blood pressure, and total cholesterol	Shift work -> SARS-CoV-2: OR=0.52 (95%-CI 0.34-0.80)
Zuñiga et al. 2022 (20)	Logistics regression. A separate model was fitted for regions with <4%, 4-8%, and >10% seropositivity	Age, gender, sector (hospital vs. primary care), workplace (emergency services, non-emergency patient care, and non-patient facing-services), profession, contact with a COVID-19 case at work or outside of work, COVID-19 symptoms, use of PPE, use of public transport, and comorbidities including tobacco use	Night-shift work -> SARS-CoV-2: OR=1.71 (95%-CI 1.41-2.07) in regions <4% seropositivity, OR=1.58 (95%-CI 1.43-1.74) in regions 4-8% seropositivity, OR=1.58 (95%-CI 1.45-1.72) in regions >10% seropositivity

Table S4. Results of meta-analysis and subgroup analyses of the association between night-shift work and SARS-CoV-2 infection. [CI: confidence interval; OR: odds ratio]

		**Studies (n)**	**Groups within studies (n)**	**OR (95%-CI)**	**I^2 (%)**	**Meta-regression OR (95%-CI)**
SARS-CoV-2	Overall	10	15	1.31 (1.09-1.58)	92.2	
Study design	Prospective cohort study	3	4	1.74 (1.47-2.06)	38.3	1.48 (1.03-2.14)
	Cross-sectional study	7	11	1.17 (0.94-1.46)	93.2	Reference
Risk of bias	High	2	2	1.14 (0.23-5.74)	91.7	Reference
	Moderate	5	7	1.36 (1.04-1.78)	88.8	1.46 (0.74-2.89)
	Low	3	6	1.37 (1.14-1.65)	87.1	1.46 (0.74-2.89)
Time	Before April 2021	6	10	1.51 (1.26-1.81)	90.8	Reference
	From April 2021 onwards	4	5	0.92 (0.68-1.26)	74.4	0.61 (0.43-0.87)
Measurement of outcome	Self-report	7	9	1.04 (0.85-1.27)	79.9	Reference
	Serology or PCR tests	3	6	1.63 (1.54-1.72)	0.0	1.65 (1.37-2.00)
Occupational class	Healthcare workers	4	7	1.46 (1.14-1.88)	94.5	1.25 (0.86-1.83)
	Other workers	6	8	1.17 (0.88-1.56)	85.9	Reference

Table S5. Summary of findings including quality of the evidence based on GRADE. [CI: confidence interval; GRADE: Grading of Recommendations, Assessment, Development and Evaluations; OR: odds ratio]

	Outcome	
	**Common respiratory infections**	**SARS-CoV-2**
Risk of bias	Serious risk of bias[a]	Serious risk of bias[a]
Inconsistency	Serious inconsistency[b]	Serious inconsistency[b]
Indirectness	No serious indirectness[c]	No serious indirectness[c]
Imprecision	Serious imprecision[d]	No serious imprecision[d]
Publication bias	No serious publication bias[e]	No serious publication bias[e]
Number of participants (studies)	43,118 (4 studies)	148,170 (10 studies)
Relative effect (95%-CI)	OR=1.11 (0.97-1.27)	OR=1.31 (1.09-1.58)
Quality of the evidence (GRADE)	Very low +	Very low +

[a] Most studies were cross-sectional studies. For common respiratory infections only 1 prospective cohort study was available and for SARS-CoV-2 infection only 3 prospective cohort studies were available. In addition, only 2 out of 4 studies on common respiratory infections and 4 out of 10 studies on SARS-CoV-2 infection adjusted for occupation or other important work-related infection exposure variables. Thus, there is serious risk of bias for both outcomes.
[b] Based on the I^2, there is substantial heterogeneity in the meta-analysis on common respiratory infections (65.8%) and high heterogeneity in the meta-analysis on SARS-CoV-2 infection (92.2%). Thus, there is serious inconsistency for both outcomes.
[c] Population, Exposure, Control, Outcome (PECO outline) were consistent with the research question of the systematic review. Thus, there is no serious indirectness for both outcomes.
[d] The meta-analysis for common respiratory infections is based on only 4 studies and the confidence interval includes the null value. The meta-analysis for SARS-CoV-2 is based on 10 studies and the confidence interval does not include the null value. Thus, there is serious imprecision for common respiratory infections, but no serious imprecision for SARS-CoV-2 infection.
[e] The results of the funnel plot, Egger's test, and trim-and-fill method do not indicate serious publication bias for both outcomes. Thus, there is no serious publication bias for both outcomes.

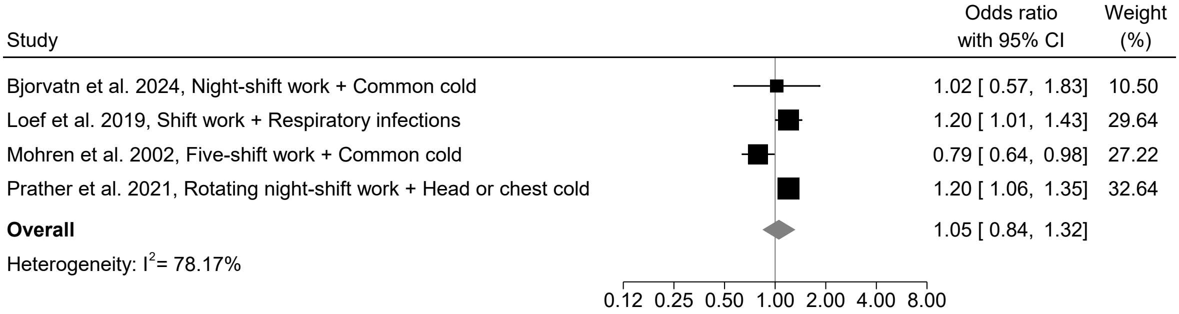

Figure S1. Forest plot of the association between night-shift work and common respiratory infections, including only one result per study by selecting the result based on the largest sample size and for one common respiratory infection outcome per study. CI: confidence interval.

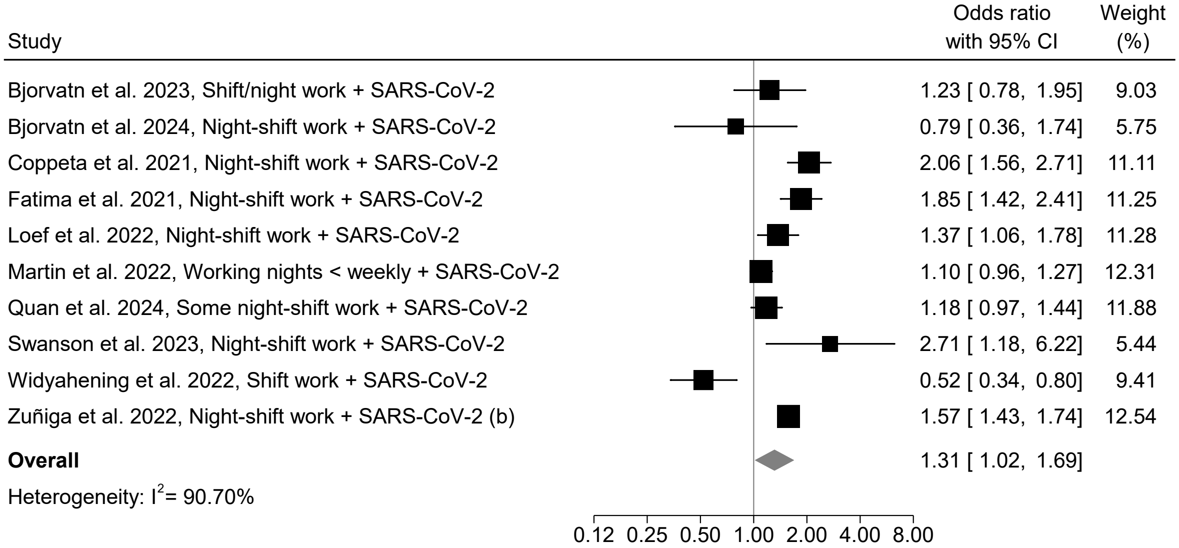

Figure S2. Forest plot of the association between night-shift work and SARS-CoV-2 infection, including only one result per study by selecting the result based on the largest sample size. CI: confidence interval. In the study of Zuñiga et al. 2022, due to significant variations in the rate of seropositivity in the various regions of Chile, regions were grouped into three categories: a) regions with <4% seropositivity, b) regions with 4-8% seropositivity, and c) regions with >10% seropositivity. Group b had the largest sample size and was therefore included in this sensitivity analysis.

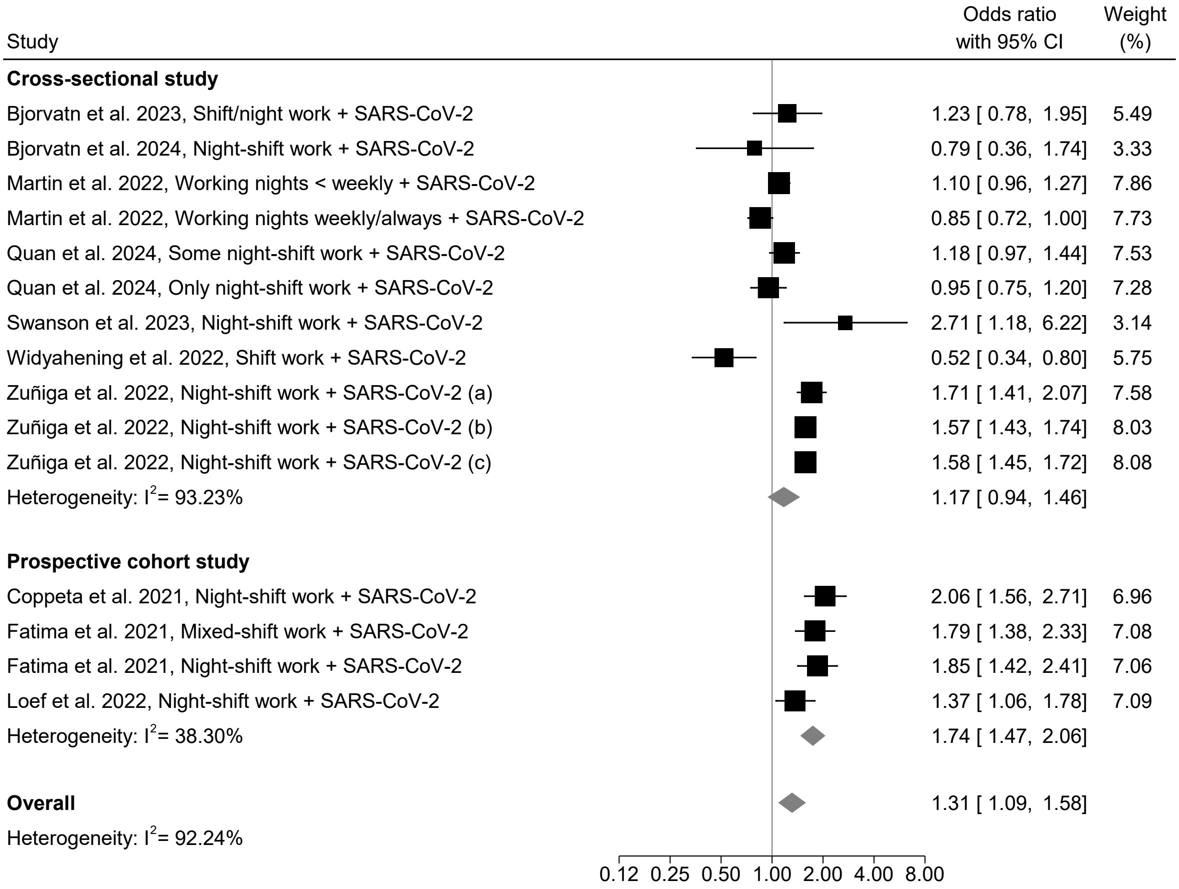

Figure S3. Forest plot of the association between night-shift work and SARS-CoV-2 infection, stratified by study design. CI: confidence interval. In the study of Zuñiga et al. 2022, due to significant variations in the rate of seropositivity in the various regions of Chile, regions were grouped into three categories: a) regions with <4% seropositivity, b) regions with 4-8% seropositivity, and c) regions with >10% seropositivity.

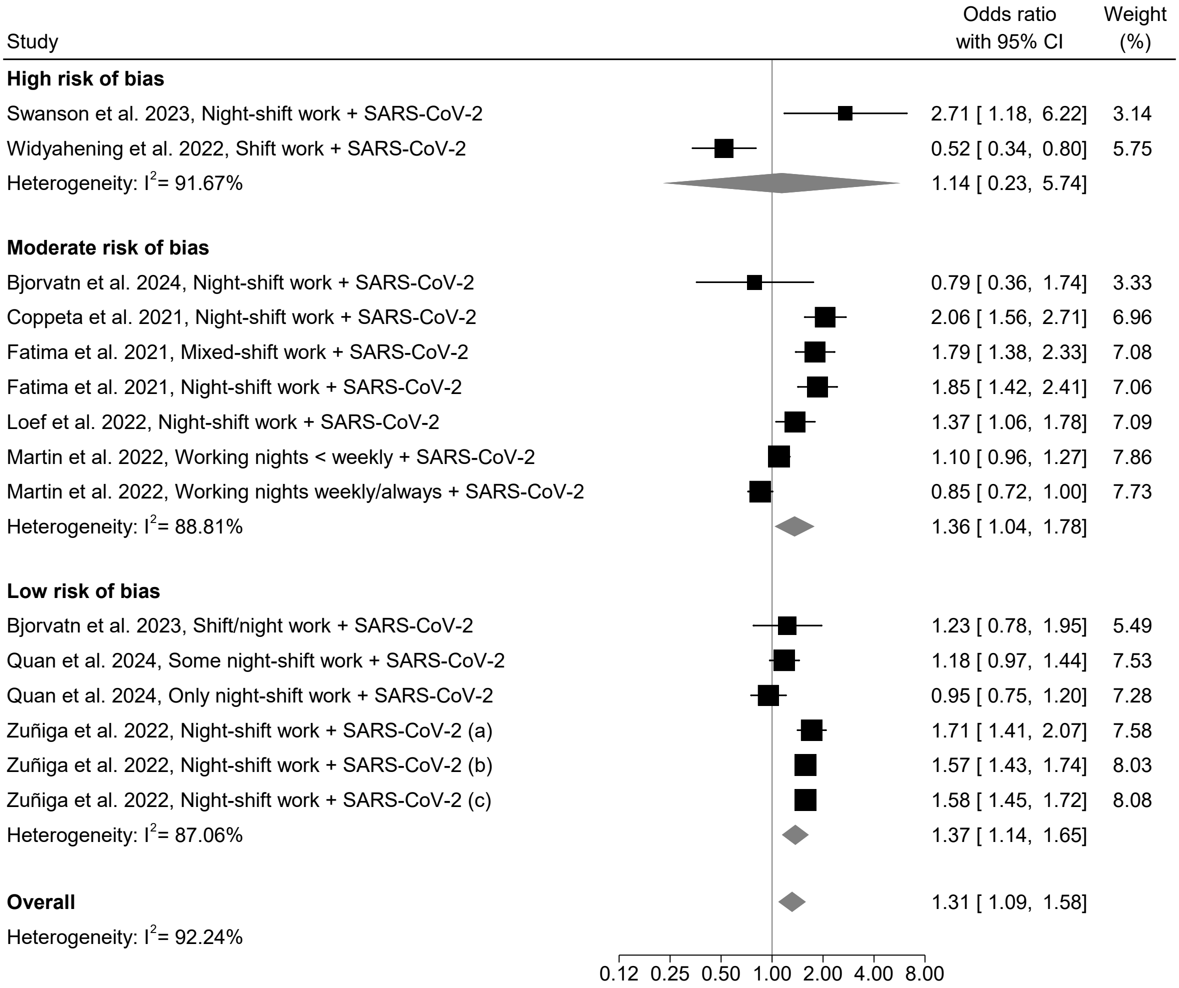

Figure S4. Forest plot of the association between night-shift work and SARS-CoV-2 infection, stratified by risk of bias. CI: confidence interval. In the study of Zuñiga et al. 2022, due to significant variations in the rate of seropositivity in the various regions of Chile, regions were grouped into three categories: a) regions with <4% seropositivity, b) regions with 4-8% seropositivity, and c) regions with >10% seropositivity.

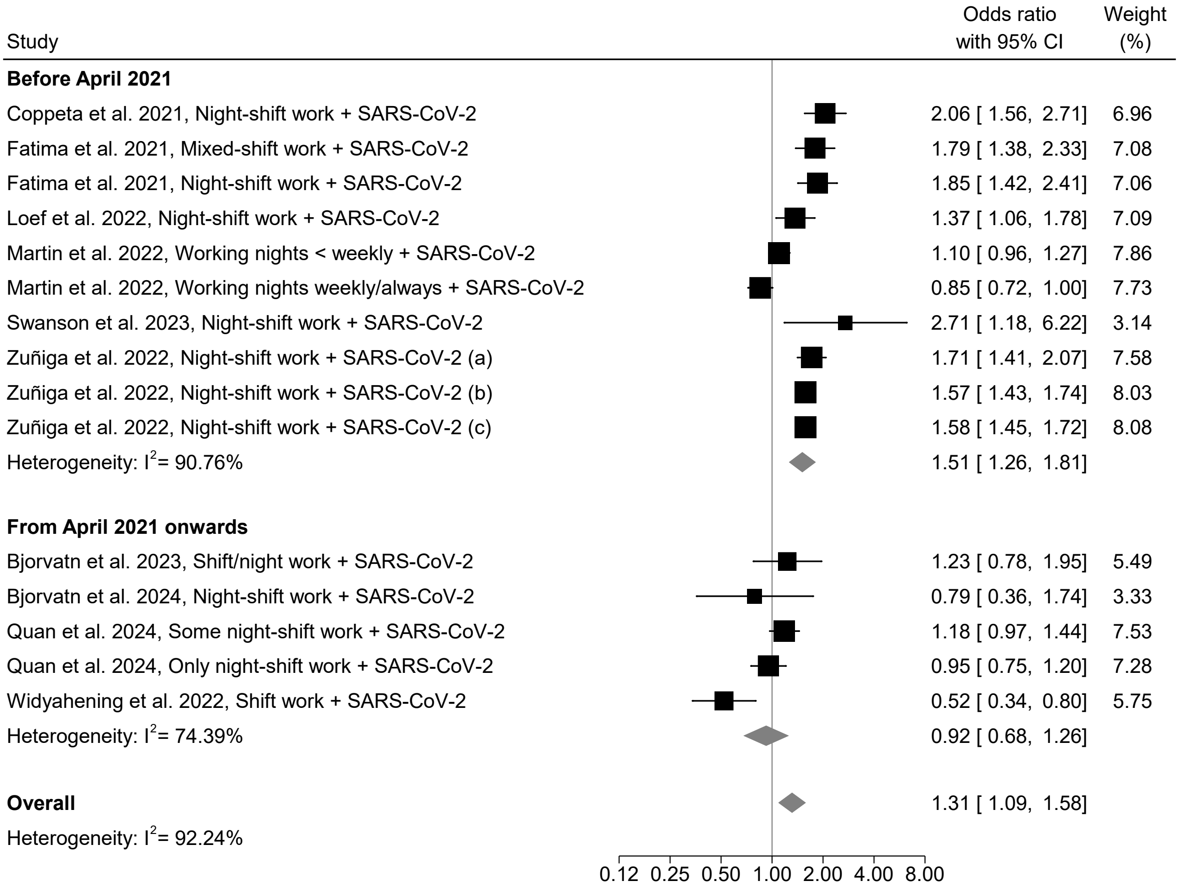

Figure S5. Forest plot of the association between night-shift work and SARS-CoV-2 infection, stratified by time. CI: confidence interval. In the study of Zuñiga et al. 2022, due to significant variations in the rate of seropositivity in the various regions of Chile, regions were grouped into three categories: a) regions with <4% seropositivity, b) regions with 4-8% seropositivity, and c) regions with >10% seropositivity.

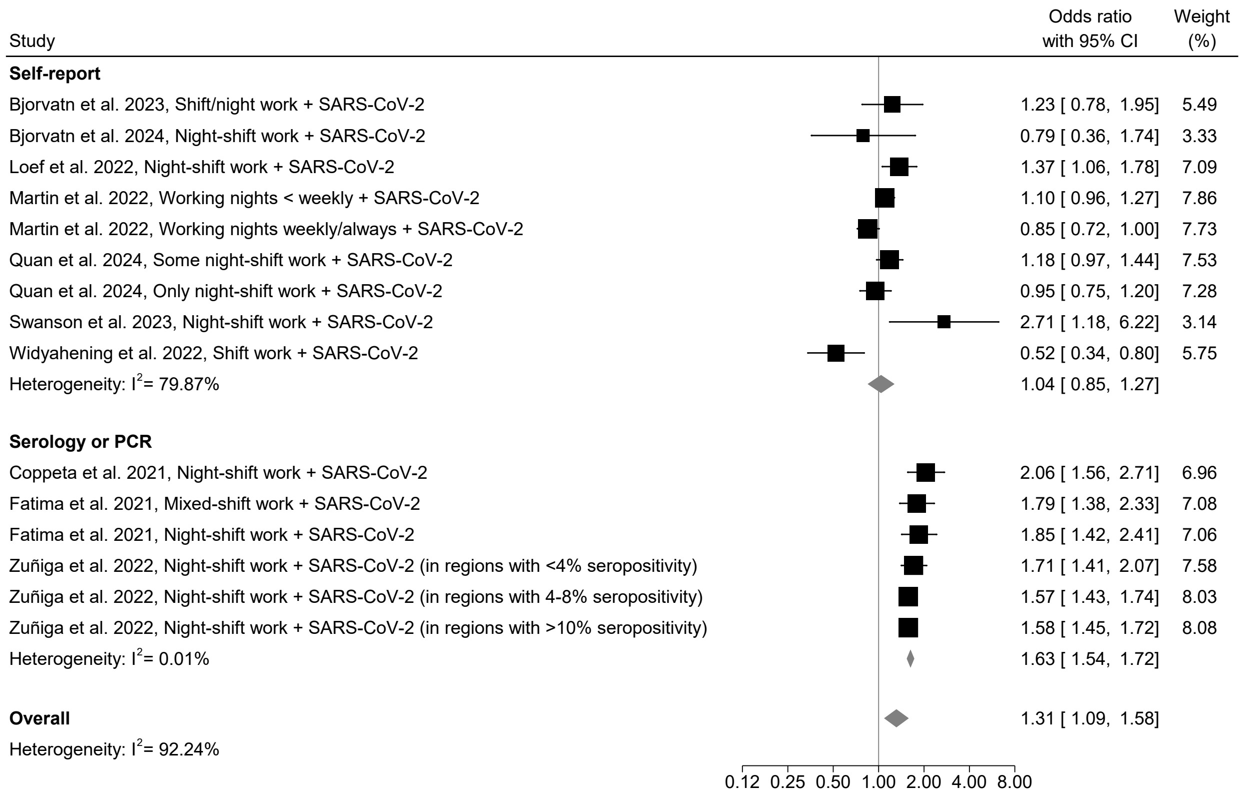

Figure S6. Forest plot of the association between night-shift work and SARS-CoV-2 infection, stratified by measurement of outcome. CI: confidence interval. In the study of Zuñiga et al. 2022, due to significant variations in the rate of seropositivity in the various regions of Chile, regions were grouped into three categories: a) regions with <4% seropositivity, b) regions with 4-8% seropositivity, and c) regions with >10% seropositivity. In Zuñiga et al. 2022, SARS-CoV-2 infection was assessed by serology in combination with self-report.

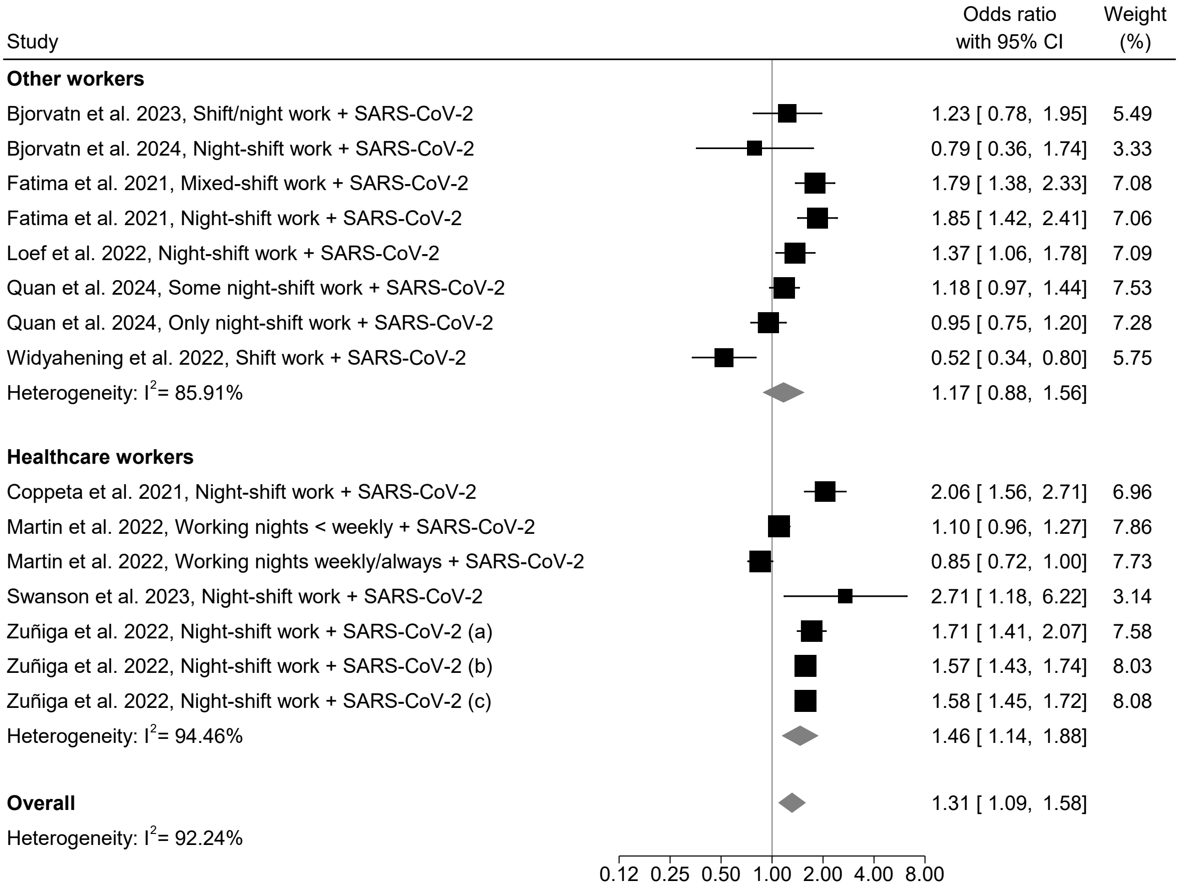

Figure S7. Forest plot of the association between night-shift work and SARS-CoV-2 infection, stratified by occupational class. CI: confidence interval. In the study of Zuñiga et al. 2022, due to significant variations in the rate of seropositivity in the various regions of Chile, regions were grouped into three categories: a) regions with <4% seropositivity, b) regions with 4-8% seropositivity, and c) regions with >10% seropositivity.

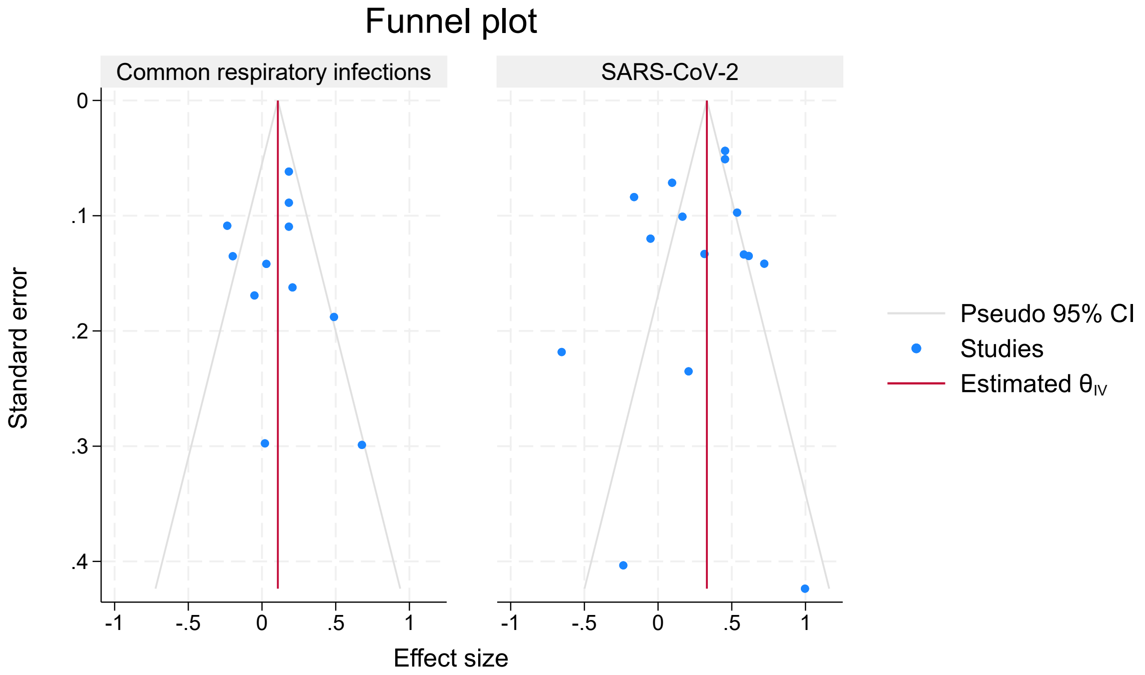

Figure S8. Funnel plot for the association between night-shift work and common respiratory infections, and night-shift work and SARS-CoV-2 infection

Text S1. Newcastle-Ottawa quality assessment Scale (NOS) for cohort studies

Selection (maximum 4 stars(*))
1) Representativeness of the night-shift worker cohort
a) Truly representative of the average night-shift worker in the target population (all subjects or random sampling) *
b) Somewhat representative of the average night-shift worker in the target population (non-random sampling) *
c) Selected group of users, e.g. population from only 1 organization
d) No description of the derivation of the cohort

2) Selection of the non-shift worker cohort
a) Drawn from the same community as the night-shift worker cohort *
b) Drawn from a different source
c) No description of the derivation of the non-shift worker cohort

3) Ascertainment of night-shift work
a) Secure record (e.g. work schedule records) *
b) Structured interview *
c) Written self-report
d) No description

4) Demonstration that infectious disease of interest was not present at start of study
a) Yes *
b) No

Comparability (maximum 2 stars)
1) Comparability of night-shift and non-shift worker cohorts on the basis of the design or analysis
a) Study controls for occupation or other important work-related infection exposure variable *
b) Study controls for any additional factor (i.e. a sociodemographic factor such as age or education, or vaccination status, or infectious disease exposure at home) *

Outcome (maximum 3 stars)
1) Assessment of outcome
a) Independent blind assessment *
b) Record linkage *
c) Self-report
d) No description

2) Was follow-up long enough for outcomes to occur
a) Yes 1 month (infectious diseases can occur immediately, so follow-up time does not need to be long if the population is sufficiently large. For most infectious diseases the incubation periods range from a few days to a few weeks, so we set this follow-up time to be at least 1 month) *
b) No

3) Adequacy of follow up of cohorts
a) Complete follow up - all subjects accounted for *
b) Subjects lost to follow up unlikely to introduce bias - small number lost -> 80% follow up or description provided of those lost *

c) Follow up rate <80% (select an adequate %) and no description of those lost
d) No statement

Text S2. Newcastle-Ottawa quality assessment Scale (NOS) for cross-sectional studies (adapted from Herzog et al. 2013)

Selection (maximum 4 stars (*))
1) Representativeness of the sample:
a) Truly representative of the average night-shift worker in the target population (all subjects or random sampling) *
b) Somewhat representative of the average night-shift worker in the target population (non-random sampling) *
c) Selected group of users, e.g. population from only 1 organization
d) No description of the sampling strategy

2) Sample size:
a) Justified and satisfactory *
b) Not justified, but satisfactory (≥100 participants per independent variable) *
b) Not justified or not satisfactory

3) Non-respondents:
a) Comparability between respondents and non-respondents characteristics is established, and the response rate is satisfactory (≥50%) *
b) The response rate is unsatisfactory (<50%), or the comparability between respondents and non-respondents is unsatisfactory
c) No description of the response rate or the characteristics of the responders and the non-responders

4) Ascertainment of night-shift work (risk factor):
a) Validated measurement tool *
b) Non-validated measurement tool, but the tool is available or described*
c) No description of the measurement tool

Comparability (maximum 2 stars)
1) The subjects in different outcome groups are comparable, based on the study design or analysis. Confounding factors are controlled.
a) The study controls for occupation or other important work-related infection exposure variable *
b) The study control for any additional factor (i.e. a sociodemographic factor such as age or education, or vaccination status, or infectious disease exposure at home) *

Outcome (maximum 3 stars)
1) Assessment of infectious disease (outcome):
a) Independent blind assessment **
b) Record linkage **
c) Self report *
d) No description

2) Statistical test:
a) The statistical test used to analyze the data is clearly described and appropriate, and the measurement of the association is presented, including confidence intervals or the probability level (p value) (Appropriate: regression analysis with confidence intervals) *
b) The statistical test is not appropriate, not described or incomplete
